# Perspectives on power relations in human health and well-being

**DOI:** 10.1080/17482631.2017.1358581

**Published:** 2017-08-23

**Authors:** Ingrid Larsson, Henrika Jormfeldt

**Affiliations:** ^a^ School of Health and Welfare, Halmstad University, Halmstad, Sweden

As guest editors of the thematic cluster Perspectives on Power Relations in Human Health and Well-being in the *International Journal of Qualitative Studies on Health and Well-being*, we would like to introduce the theme related to the five included articles. The incorporated papers discuss aspects of power in different contexts and power’s effects on health and well-being in human relations. Power in human relationships is, among other things, related to gender, age, and differences in knowledge about health issues as well as to contextual factors. The contexts of the studies included range from sports clubs for young people and personal trainers’ (PTs) coaching of individuals regarding health issues to football coaches managing interpersonal relationships with their players in order to win competitions. The cluster also comprises research regarding healthy habits in different populations, and ethical issues related to research among individuals with severe mental health problems such as schizophrenia. In common for all included articles is that they concern power relations related to human health and well-being.

All human beings have equal worth regardless of their gender, age, background, or disability, and persons possessing any kind of a professional coaching function should treat other human beings with respect (UN General Assembly, 1948). The concept of *Homo capax* has developed from the perspective that every human being is a capable person with inherent power, capacity, and resources. Capable persons have the ability to speak, act, and take responsibility for their actions. Each person is both an acting individual and a suffering individual depending on the life events that they are experiencing. Human beings are striving for the good life characterized by harmony and by mutual respect for others (Ricoeur, 2011). In the area of health and well-being, focus on interpersonal relations is critical. In order to promote human freedom, every relationship must be built on respect for the inherent value of each person and a willingness to support each person’s strengths and abilities, and interpersonal relations have to be built on mutual respect for each other’s knowledge and life experiences (Morgan & Yoder, 2012). Hence, it is important to take into account the whole person and his or her physical, psychological, social, and spiritual needs. The importance of seeing all individuals as capable persons is described by Lindgren, Annerstedt, and Dohlsten (2017), who explore what sport clubs could do to retain their young adult members. Focus-group interviews were conducted with 27 coaches and 28 young adults. In this grounded theory, the core category “The individual at the centre of a community” summarized a process in which the coaches pay attention to the young adults’ needs and interests regardless of their backgrounds and skills. Three main categories reflected key components in sports clubs: participation and influence, including having their voices heard and participating on equal terms; social connectedness, including friends and fellowship and emotional support and security; and good conditions reflected in low costs and the development of facilities (Lindgren et al., 2017). Moreover, Lindgren and Barker-Ruchti (2017) illuminate how a sample of female Swedish national football coaches describe how they carefully manage interpersonal relationships with respect to the well-being of their players as they face social and organizational pressure to win competitions. In-depth interviews were conducted with five female football coaches, and this resulted in the overarching theme of “a holistic perspective of every person”. Four categories revealed that female football coaches focus on promoting development, well-being, and sustainability, listening to feelings of demands and expectations, managing challenging behaviour, and promoting fair play for the greater good (Lindgren & Barker-Ruchti, 2017).

Knowledge and power are concepts that affect each other because knowledge creates power and power increases through knowledge (Kuokkanen & Leino-Kilpi, 2000). All human relationships thus incorporate power relations in different contexts (Bradbury-Jones, Sambrook, & Irvine, 2008; Kuokkanen & Leino- Kilpi, 2000). Global health—like the arts, law, and medicine—is a social arena with specific rules, but one where people hold unequal positions (Shiffman, 2015). In the area of healthcare, providers have more power and expertise than clients (Brown, 2016), and in contexts that incorporate older people, unequal power relations are common (Eliassen, 2016). There are also aspects of power involved in the relationship between the researcher and the participant in both quantitative and qualitative research. The researcher has the power to determine the research questions and the methods of data collection and analysis, but the researcher also has the opportunity to share power with the participant (Florczak, 2016; Karnieli-Miller, Strier, & Pessach, 2009). Carlsson, Blomqvist, and Jormfeldt (2017) argue that involving people with schizophrenia in research is critical to promoting health and well-being among this target group and that the quality of qualitative research needs to be scrutinized for methodological issues. Fifteen qualitative interview studies involving people with schizophrenia were critically reviewed with respect to methodological issues related to ethics, data collection, and analysis of data in order to find ways to reinforce the power of people with schizophrenia (Carlsson et al., 2017).

Sharing knowledge and expertise between persons is a key component of mutuality. Mutuality is often described as interdependence and influence in relationships with other people in different contexts, and the concept also includes power and social capital. Social capital, including relationships, networks, and available resources, is an essential aspect of power that people need in order to manage their lives. The perspective of social capital as a resource in a person’s life might enable new ways of empowering people (Brown, 2016). Jonsson, Larsson, Berg, Korp, and Lindgren (2017) investigate factors that undermine healthy habits with regard to exercise and diet among adolescents in a disadvantaged community. Focus-group interviews conducted with 53 adolescents revealed that there are several factors at the individual, social, environmental, and societal levels that undermine adolescents’ chances of establishing healthy exercise and dietary habits. Temptations challenging a healthy lifestyle among young people were found, such as screen-based activities and emotional eating. The adolescents in the study also described a lack of support from the surrounding environment. Norms and demands from others also set the agenda for adolescents. Boys described how they were expected to participate in physical activities, while girls stated that they were expected to do more domestic work than boys at home (Jonsson et al., 2017). Håman, Lindgren, and Prell (2017) illuminate PTs’ understanding of healthy and unhealthy exercise and eating behaviours in relation to orthorexia nervosa in a fitness gym context. Focus-group interviews were conducted with 14 PTs and analysed using firstly an inductive and secondly a deductive method according to the four types of deviant behaviour—pure, conforming, falsely accused, and secret (Becker, 1997). In orthorexia, pure deviation behaviour was related to excessive and controlling behaviours, conforming behaviour was related to balanced behaviour and contributed to well-being, falsely accused behaviour was related to extreme behaviours in line with a forceful exercise trend, and secret deviant behaviour was related to healthy behaviours with an unhealthy mind (Håman et al., 2017). An important aspect of health in young people is for them to receive sufficient support to gain the power needed to influence their own life in a healthy way. Empowerment can be defined both as a goal, to have control over the determinants of a person’s quality of life, and as a process, to take control over the change process (Tengland, 2008). Empowerment defined as a process comprises personal growth and development, with the main factors being a person’s beliefs, views, values, and relationships with other people. Empowerment includes a positive self-identity and an ability to interact with other people (Kuokkanen & Leino-Kilpi, 2000). Interactions with other people incorporate power relations and establish ways of getting things done (Brown, 2016). Empowered persons experience themselves as capable persons; for example, they feel they have the power to do things in a good, effective, and constructive way (Kuokkanen & Leino-Kilpi, 2000), and this includes areas such as health and well-being.

Even though all of the included articles do not explicitly express a focus on power relations, aspects of power are intrinsically interwoven into all of the relationships between the people targeted in the papers. The effects of these power relations regarding the health and well-being of the individuals involved should be clear to the reader if awareness of the phenomenon is consciously regarded. We hope that this thematic cluster will supply the reader with a new and widened understanding of power relations and deepen their insight into the importance of power relations in health and well-being.
